# Suicide attempts, self-harm and suicidal ideation among Norwegian university students with eating disorders and disordered eating: the SHoT study 2018 and 2022

**DOI:** 10.1186/s12888-026-08229-0

**Published:** 2026-05-30

**Authors:** Lisa Marie Jacobsen, Gørill Haugan, Scott Patten, Tonje Braaten, Børge Sivertsen, Ottar Bjerkeset

**Affiliations:** 1 Faculty of Nursing and Health Sciences, Nord University, Levanger, Bodø, Norway; 2 Department of Community Health Sciences, Department of Psychiatry, University of Calgary, Calgary, Canada; 3 Cuthbertson & Fischer Chair of Pediatric Mental Health, University of Calgary, Calgary, Canada; 4 Department of Community Medicine, UiT The Arctic University of Norway, Tromsø, Norway; 5 Department of Health Promotion, Norwegian Institute of Public Health, Bergen, Norway; 6 Department of Research and Innovation, Helse Fonna HF, Haugesund, Norway

**Keywords:** Eating disorders, Self-harm, Suicide attempts, Suicidal ideation, Disordered eating, EDS-5, Emerging adults, Post-secondary students, Anorexia nervosa, Bulimia nervosa, Binge eating disorder

## Abstract

**Background:**

Eating disorders (EDs) and disordered eating (DE) are associated with elevated risk for suicide attempts, self-harm and suicidal ideation. Still, gender-specific knowledge about the frequency and association of EDs and DE with these outcomes among university students is limited. This study examines gender-stratified frequencies and associations between EDs and DE, and suicide attempts, self-harm and suicidal ideation.

**Methods:**

We pooled data from the students` Health and Wellbeing Study (SHoT) collected in 2018 and 2022, including Norwegian fulltime students aged 18–35 (N: 98 925). Participants completed self-report measures assessing EDs (including subtypes) and symptoms of DE (EDS-5), suicide attempts, self-harm and suicidal ideation during the last 12 months. Statistical analyses included logistic regression to assess associations between EDs, DE and suicide attempts, self-harm, and suicidal ideation. Further, a single item analysis of the EDS-5 was performed for the same outcomes.

**Results:**

We found that female, male and gender diverse students with EDs had substantially elevated odds of suicide attempts, self-harm and suicidal ideation, and for DE, moderately elevated odds, compared to students without these conditions. We found the strongest associations among males with EDs, but precision was limited in several subgroup analyses because of small cell counts. Particularly items “comfort eating” and “feeling guilty about eating” on EDS-5 were closely associated with reports of suicide attempts, self-harm and suicidal ideation.

**Conclusions:**

We found consistently high rates of suicide attempts, self-harm and suicidal ideation among students with EDs and DE across genders, and robust associations between EDs and DE, and suicide attempts, self-harm and suicidal ideation. These findings underscore the importance of considering gender-specific patterns and targeted items to enhance assessment practices in student health settings.

**Trial registration:**

Identifier: NCT05731102.

**Supplementary Information:**

The online version contains supplementary material available at 10.1186/s12888-026-08229-0.

## Background

Eating disorders (EDs) are characterized by preoccupation with body weight and shape, and dysfunctional eating patterns [1, 2]. In Western countries, the prevalence has been estimated to about 2–3% in females and less than 1% in males [3]. Particularly, the transition from adolescence to adulthood is a vulnerable period for developing EDs [4] and other mental health problems and disorders [5]. These include suicide attempts, self-harm and suicidal ideation [6, 7], and common mental disorders like anxiety and depressive disorders [8], all major public health concerns serving as important markers of mental distress as well as predictors of future suicide [9]. Among individuals with anorexia nervosa (AN), medical complications are the leading cause of death, followed by suicide [10]. Self-harm and suicidal behaviours arise from interactions between predictors [11], such as gender, psychological distress, socioeconomic status, physical health, early life neglect and abuse, bullying, loneliness and financial situation [6, 9, 12, 13].

One-quarter to one-third of adolescents and adult patients with AN, bulimia nervosa (BN), or binge eating disorder (BED) report suicidal ideation, and similar proportions of patients with AN or BN have attempted suicide [14]. Further, self-harm is associated with lower recovery rates in EDs [15]. Findings from a systematic review and meta-analysis indicate that ED populations have an increased prevalence of suicide attempts, self-harm and suicidal ideation compared to psychiatric controls (including major depressive disorder, personality disorder, bipolar disorder, and other non-psychotic disorders) [16]. A meta-analysis of 32 334 individuals with EDs confirmed that those with AN and BN reported the highest prevalence of self-harm [17]. Another large systematic review and meta-analysis, including 29 studies from 11 countries, found that about one in three ED patients reported life-time prevalence of self-harm [18].

Disordered Eating (DE) is common in young people and concerning from a public health perspective, highlighting the need to implement strategies for preventing EDs [19]. DE is seen as a part of a continuum and present an “at risk” status [20, 21], often used when symptoms of abnormal eating are present but not reaching the threshold of an ED diagnosis [22]. DE associates with mental distress [23] and has been linked to later EDs [24, 25]. A Norwegian study on more than 7 000 adolescents found an association between suicidal ideation and DE, and especially in males [26], while a UK study on students found a co-occurrence of DE and self-harm in 4.5–4.9% of the students [27].

Students with EDs often report self-harm and suicidal behaviors accompanied by increased risk of academic failure [28]. Research based on The students Health and Well-being Study (SHoT) documented increasing levels of self-reported mental health problems [29] and suicide attempts, self-harm and suicidal ideation among university students [30, 31], and an overall increasing ED prevalence [32] from 2018 to 2022. Further, a Swedish study of more than 3 000 students found that 50% of those with EDs reported lifetime self-harm, and as many as 70% reported lifetime suicide ideation [33]. US research on EDs among post-secondary students also confirms the strong association between DE and self-harm [34]. A large-scale study including more than 400 000 American college students between 2015 and 2019 identified substantial variation in ED prevalence across gender identities, sexual orientations, and racial and ethnic groups [35]. Among gender diverse youth, research consistently show even higher rates of EDs [32], DE and suicidal ideation and -attempts compared to their cisgender peers [36–38].

The associations between EDs, DE and self-harm and suicidal behaviors are well documented in clinical samples [10, 16–18, 39]. To date, though, few large epidemiological studies have examined gender-specific associations of EDs and DE with self-harm and suicidal behavior among university students. Yet this knowledge is needed, so that universities and student health services can implement the most relevant support and mental health services for this vulnerable group of students.

In this study, we first describe the characteristics of Norwegian students with self-reported EDs (AN, BN and BED) and DE. The main aim is to estimate the proportion of students with EDs and DE who have experienced suicide attempts, self-harm or suicidal ideation in the last 12 months, by gender (female, male and gender diverse). Finally, we examined the association between each item of the EDS-5 scale and suicide attempts, self-harm or suicidal ideation last 12-months.

## Methods

### Study design and setting

**Fig. 1:**
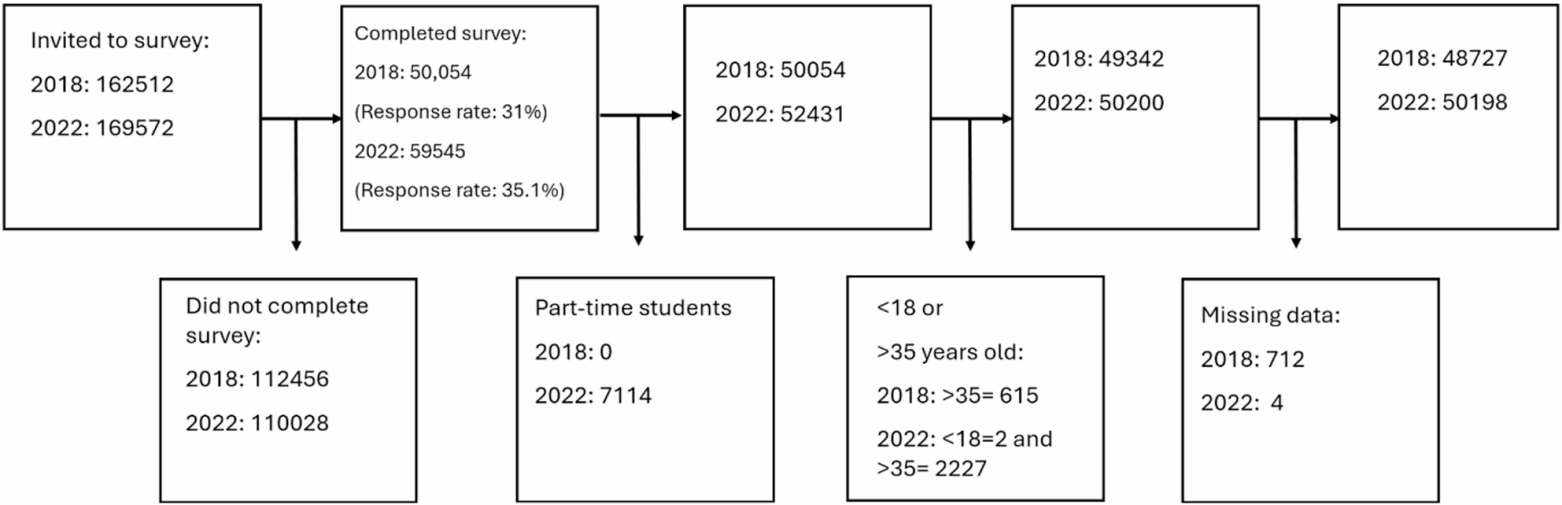
Flow chart of inclusion of students

SHoT is a repeated cross-sectional survey among all Norwegian university fulltime students aged 18–35, carried out electronically through a web-based platform [29]. We used data from 2018 to 2022. Detailed information about SHoT has been published elsewhere [40]. SHoT 2018 was conducted between February 6 and April 5, 2018. Among 162 512 students invited, 50 054 students completed the survey (response rate: 31%) [41] (Fig. 1). SHoT 2022 was carried out between February 6 and April 19, 2022, and a total of 169 572 students were invited and 59 545 completed (response rate: 35.1%) [42]. Students without recorded age were excluded from the analysis and categorized as missing data (Fig. 1).

### Outcome variables

In SHoT, suicide attempts, self-harm and suicidal ideation were self-reported with three items drawn from the Adult Psychiatric Morbidity Survey [43]; *“Have you ever seriously thought of taking your life*,* but not actually attempted to do so?“; “Have you ever made an attempt to take your life*,* by taking an overdose of tablets or in some other way?“; “Have you ever deliberately harmed yourself in any way but not with the intention of killing yourself? (i.e.*,* self-harm)“.* If yes to any item, the timing of the most recent episode was assessed, using the following response options: *“last week”*,* “past year”*,* “more than a year ago*,* but after I started studying at the university”*, and *“before I started studying at university”.* In this study we only included self-harm and suicidal behaviours occurring in the past year; responses were dichotomized as “*yes*” or “*no*” [44].

### Exposure variables

EDs were assessed using a self-report comprehensive survey instrument that listed a range of mental and somatic health conditions, asking participants if they had experienced any illnesses or disorders in the past 12 months. If *“mental disorders”* were selected, a follow-up list of diagnostic categories were presented, including *“Eating disorders”* as an option (further called any ED). In addition, they could specify subtypes such as “Anorexia,” “Bulimia,” or “Binge eating disorder” [45]. ED subtypes do overlap and were therefore not categorized as mutually exclusive categories in this study. Hence, it was possible for students to self-report more than one ED diagnosis.

The eating disturbance scale (EDS-5) is a 5-item self-report questionnaire developed by Rosenvinge and colleagues, and measures disturbed eating patterns, validated on Norwegian students (mean age 25.2) [46]. The Cronbach alpha was 0.83 and 0.86, and sensitivity and specificity were 0.90 in females and 0.88 in males with respect to DSM-IV EDs [46]. The EDS-5 focuses on ED behavioural characteristics during the last month, and is scored on a Likert-scale from 1 to 7 (1=“never” – 7=“every day” or 1=“very satisfied” or 7= “very dissatisfied”) [46]: *“Are you satisfied with your eating habits?”*, “*have you eaten to comfort yourself because you were unhappy?”*, “*have you felt guilty about eating?”*, “*have you felt that it was necessary for you to use a strict diet or other eating rituals to control your eating?”*, and “*have you felt you are too fat?”.* The responses were dichotomized into 0 (scores 1 through 5) and 1 (scores 6 or 7). Lastly, an EDS cut off > 4.1 was used (DE positive) according to a previous study among students [47]. Given limited empirical validation of this threshold, we additionally evaluated its performance using receiver operating characteristic (ROC) analyses. Self-reported ED (any ED) served as the reference standard in the ROC analyses.

### Age and gender

Age was extracted from a National Register using the participants’ National 11-digit identity number. In SHoT 2018 the students were asked if they identified as “*female”*,* “male”*,* “trans”*, or *“others”.* In 2022, “*female”*,* “male”*,* “non-binary”* or “*cross-dressers/others/I don’t know”* were the response options. Those who did not identify as either female or male were combined into a gender diverse group.

**Mental health problems** were assessed by the widely used *Hopkins Symptoms Checklist* (HSCL-25) derived from the 90-item Symptom Checklist (SCL-90) [48], to assess symptoms of anxiety and depression in the last 14 days. The HSCL-25 has shown high validity among Norwegian university students [49], and was dichotomized using a cut-off score of > 2 [40, 50].

### Statistical analysis

We used Stata, version 18 [51] for statistical analyses. Data from 2018 to 2022 were pooled to increase statistical power, particularly for males and gender diverse students. The 2018 and 2022 samples represent independent cross-sectional studies, with only minor overlap of participants. First, we did a descriptive analysis of the total sample and outcomes (last year suicide attempts, self-harm and suicidal ideation). Next, we stratified by any self-reported ED/no ED and DE positive/negative for the same outcomes. Before the logistic regression analyses, Mantel–Haenszel tests were used to assess heterogeneity by study year. No evidence of significant heterogeneity was identified (*p* > 0.05), suggesting that differences between survey years are consistent with sampling variability, supporting the decision to pool the two surveys. Further, we estimated age-adjusted odds ratios (ORs) with 95% confidence intervals (CIs) applying logistic regression models with outcome variables (last year suicide attempts, self-harm and suicidal ideation) and exposure variables (self-reported any ED, AN, BN, BED and DE). A complete-case analysis was conducted, excluding participants with missing data on any variables included in the model from that specific analysis. To examine whether the association between any self-reported ED and the outcomes differed by gender, we performed interaction analyses with gender as potential effect modifier using a likelihood ratio test. We also performed a logistic regression analysis of outcome variables (last year suicide attempts, self-harm and suicidal ideation) and each variable of the EDS-5, adjusted for gender and age. Finally, we performed ROC analyses to evaluate the discriminatory ability of the EDS-5 total score in identifying students with self-reported ED. Area under the curve (AUC), sensitivity, specificity, and Youden’s index were calculated. We then compared the pre-specified cut-off (> 4.1) with the ROC-derived optimal threshold to assess cut-off performance.

## Results

In total 98 925 Norwegian full-time students participated in SHoT 2018 and 2022, and about two thirds were females (Table 1). A group of 443 students (0.5%) identified themselves as gender diverse. Mean age was around 23.5 years in both cases (any self-reported ED and DE positive) and controls (no self-reported ED and DE negative). While 2.9% of the students reported any ED, 27.7% of the students were over the DE cut-off. The rates for suicide attempts, self-harm and suicidal ideation were 0.5%, 4.3% and 7.9%, respectively (Table 1).

**Table 1:** Characteristics of the total sample pooled from SHoT 2018 and 2022

Baseline sample characteristics	(*N* 98 925)
Female, %	67.4% (66 668)
Male, %	32.1% (31 681)
Gender diverse, %	0.5% (443)
Age, mean (SD)	23.5 (3.1)
**Exposure variables**	
Any ED % (N)	2.9% (2 877)
Anorexia nervosa % (N)	1.7% (1 629)
Bulimia nervosa % (N)	1.1% (1 077)
Binge eating disorder % (N)	0.9% (891)
EDS total, mean (SD)	3.2 (1.4)
Above EDS cut off % (N)	27.7% (27 265)
**Outcome variables**	
Suicide attempts last year %	0.5% (473)
Self-harm last year %	4.3% (4 236)
Suicidal ideation last year %	7.9% (7 801)

Table 2 shows that 4.1% of females, 0.5% of males and 9.9% of the gender diverse reported any ED. Within any ED group, the most common ED diagnosis was AN (56.6%), followed by BN (37.4%) and BED (31.0%). The mean EDS-score was 5.0 in the self-reported any ED group, and 3.2 among the no ED group. Among students with any ED, 3.6% reported suicide attempts, 23.7% reported self-harm and 32.5% in the past year (Table 2). Moreover, among students with any ED, 81.4% scored above the HSCL-25 cut-off, and the mean HSCL-25 was 2.9, compared to 29.7% and mean HSCL-25 1.8 in the no ED group (Table 2).

In total, 34.8% of females, 12.2% of males and 32.7% of gender diverse students were classified as DE positive. Among the DE positive students, 8.5% also reported an ED diagnosis: the most common was AN (4.5%), followed by BN (3.4%) and BED (3%). In contrast, only 0.8% of the DE negative students reported any ED. The mean EDS score was 5.0 in the DE positive group, and 2.6 in the DE negative. Among DE-positive students, 1% reported suicide attempts, 8.6% self-harm, and 14.5% suicidal ideation in the past year (Table 2). In the DE positive group, 55.5% scored above the HSCL-25 cut-off, and the mean HSCL-25 was 2.1, compared to 22% and mean HSCL-25 1.7 among the DE negative students (Table 2).

**Table 2:** Students characteristics from SHoT 2018 and 2022 combined, by ED and DE status

Baseline sample characteristics	2018 and 2022 (Total *N*: 98 925) Females: 67,4%			
	Total sample by ED status		Total sample by DE status	
	No ED 97.1% (96 048)	Any ED 2.9% (2 877)	DE negative 72.3% (70984)	DE positive 27.7% (27 236)
Female, % (N: 66 668)	95.9% (64 008)	4.1% (2 660)	64.7% (43 157)	34.8% (23 226)
Male, % (N: 31 681)	99.5% (31 515)	0.5% (166)	86.9% (27 529)	12.2% (3 865)
Gender diverse, % (N: 443)	90.1% (399)	9.9% (44)	67.3% (298)	32.7% (145)
Age, mean (SD)	23.5 (3.1)	23.2 (3.0)	23.4 (3.0)	23.6 (3.2)
HSCL-25, mean (SD)	1.8 (0.6)	2.9 (1.0)	1.7 (0.5)	2.1 (0.6)
HSCL-25 > 2 cut-off %	29.7% (27 966)	81.4% (2 308)	22.0% (15 518)	55.5% (14 756)
**Exposure variables**				
Any ED % (N)		100% (2 877)	0.8% (551)	8.5% (2 314)*
Anorexia nervosa % (N)		56.6% (1 629)	0.5% (384)	4.5% (1 236)*
Bulimia nervosa % (N)		37.4% (1 077)	0.2% (159)	3.4% (916)*
Binge eating disorder % (N)		31.0% (891)	0.1% (73)	3% (817)*
EDS total, mean (SD)	3.2 (1.3)	5.0 (1.1)	2.6 (0.8)	5.0 (0.7)
Above EDS cut off % (N)	26.0% (24 951)	80.4% (2 314)		
**Outcome variables**				
Suicide attempts last year	0.4% (369)	3.6% (104)*	0.3% (210)	1.0% (260)*
Self-harm last year	3.7% (3 553)	23.7% (683)*	2.6% (1 881)	8.6% (2 343)*
Suicidal ideation last year	7.1% (6 865)	32.5% (936)*	5.4% (3 824)	14.5% (3 946)*

*P-value < 0.05 for difference between any ED and no ED, and DE positive and DE negative

**Self-reported ED diagnoses**: It was possible to report more than one ED diagnosis, and the total % of AN, BN and BED combined therefore exceed 100%

**Missing**: Gender: 133 DE: 598 HSCL-25: 269

### Suicide attempts, self-harm and suicidal ideation among students with self-reported eating disorders

**Fig. 2:**
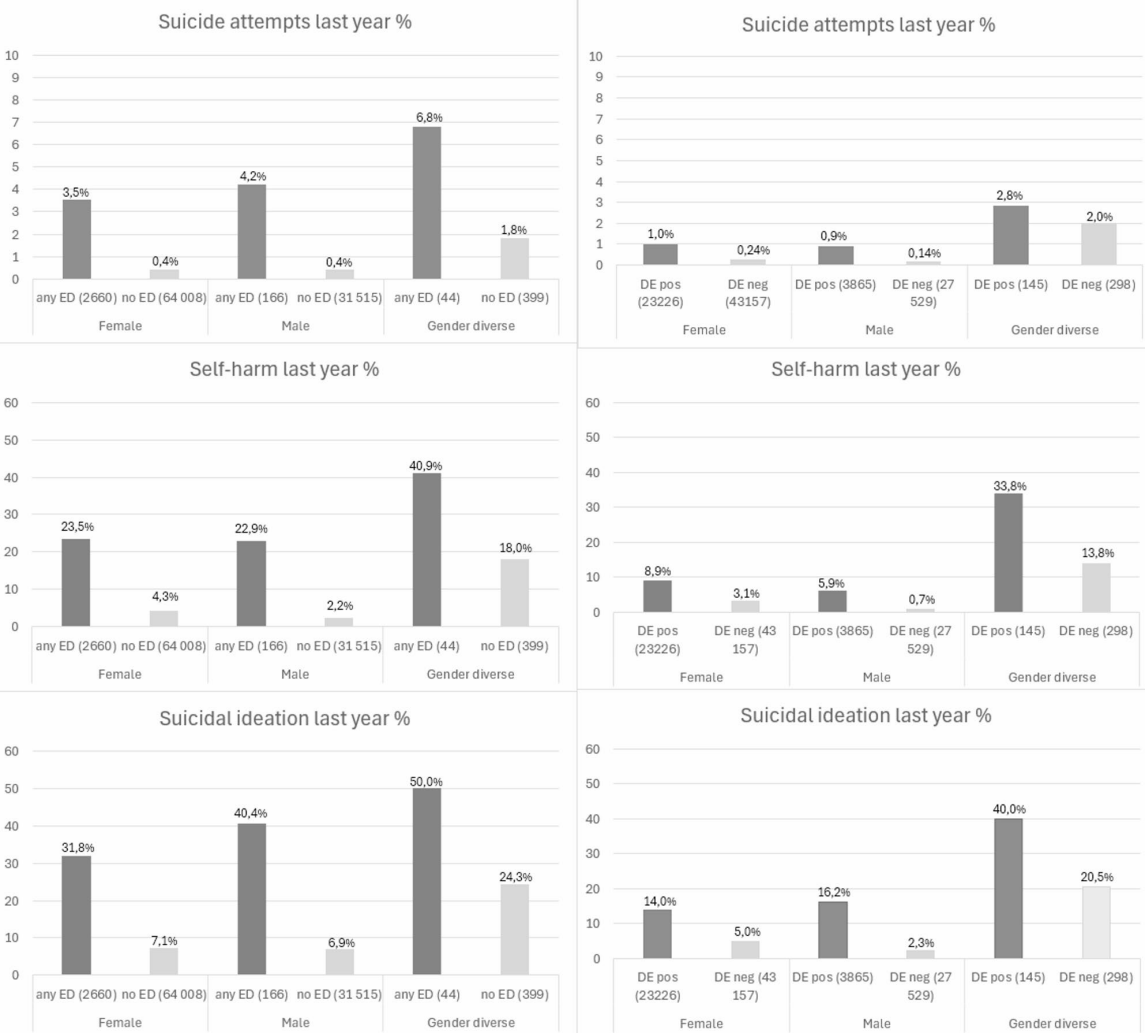
Frequency of outcomes by self-reported ED and DE-status in 2018 and 2022 (pooled data). For CIs, see appendix A2

Figure 2 shows that 3.5% of females with any self-reported ED reported suicide attempt(s) last 12 months, while 23.5% reported self-harm and 31.8% suicidal ideation. Among males, the corresponding frequencies were comparable or slightly higher: 4.2% for suicide attempt(s), self-harm 22.9% and suicidal ideation 40.4%. Lastly, the gender diverse group showed the highest proportion for all outcomes, reaching 6.8% for suicide attempts, self-harm 40.9%, and suicidal ideation 50% in the last 12 months.

For specific EDs, students with BN reported the highest suicide attempts rates among females, at 4.7%. The proportion of students reporting self-harm in females were consistent between diagnoses, ranging from 24.5% to 25.9%. For suicidal ideation among females, BN and BED had the highest prevalence rates, 34.9% and 34.2%, respectively (Appendix A2). The frequency of suicide attempts in males with self-reported AN, BN and BED was low, numbers of cases varied from zero to four. The same was the case for all outcomes among gender diverse. The self-harm rates among male students with self-report AN, BN and BED varied between 20% and 29.7%, and between 35.4% and 47.1% for suicidal ideation last year (appendix table A2).

### Suicide attempts, self-harm and suicidal ideation among students with self-reported disordered eating

After dividing the total sample into self-reported DE negative and DE positive students, we saw similar patterns as for self-reported EDs, but frequencies of the different outcomes were lower. For DE positive females, 1.0% reported suicide attempts, 8.9% self-harm and 14.0% suicidal ideation (Fig. 2). For DE positive males, 0.9% reported suicide attempts, 5.9% self-harm and 16.2% suicide ideation.

Males with BED had the highest suicide attempts prevalence, at 6.2%. (Appendix A2). For gender diverse with DE, the prevalence was 2.8% for suicide attempts, self-harm 33.8%, and suicide ideation 40.0%.

### Association of self-reported eating disorders and disordered eating with suicide attempts, self-harm and suicidal ideation

In females with EDs (any ED, AN, BN and BED) we found age-adjusted ORs from 6 to 11 for suicide attempts, self-harm and suicidal ideation (Table 3). Among male students with any ED, results showed age-adjusted ORs from 9 to 18 in all outcomes, while ORs were 3 to 6 among gender diverse students (Table 3). However, CIs were wide particularly in the two latter groups. ORs were generally higher in males than in females, yet the likelihood ratio test evaluated the interaction between any ED and all three genders (females as reference), and results showed significant interaction results in self-harm and suicidal ideation (*p* < 0.01), but not for suicide attempts (*p* = 0.42). Adjusting for anxiety and depression (HSCL-25 score) in Model 2 substantially reduced the ORs by between 25% and 70%, indicating considerable confounding effects.

Among female students with DE, we found age adjusted ORs from 3 to 4, and slightly lower estimates for male (ORs from 2.4 to 3.5) and gender diverse students (ORs 1.4 to 3.3). Yet again, numbers were low and we lack statistical evidence for an increase of suicide attempts in the gender diverse group.

**Table 3:** ORs for outcomes (95% CI) by self-reported ED/DE status in 2018 and 2022 (pooled data)

Gender	Exposure variables	Adjustment model	Suicide attempts last year	Self-harm last year	Suicidal ideation last year		
Females		Any ED	1	9.9 (7.8–12.7)	6.8 (6.2–7.5)	6.1 (5.6–6.6)	
			2	4.9 (3.8–6.3)	3.7 (3.4–4.1)	3.0 (2.8–3.3)	
		Anorexia Nervosa	1	9.6 (7.2–12.8)	6.7 (5.9–7.6)	5.6 (5.0-6.3)	
			2	4.8 (3.5–6.4)	3.6 (3.2–4.1)	2.8 (2.5–3.2)	
		Bulimia Nervosa	1	11.4 (8.4–15.6)	7.0 (6.1–8.2)	6.4 (5.6–7.3)	
			2	5.8 (4.1–7.9)	3.9 (3.4–4.6)	3.3 (2.9–3.8)	
		Binge eating disorder	1	7.0 (4.6–10.6)	6.5 (5.6–7.7)	6.1 (5.2–7.1)	
			2	3.3 (2.2–5.1)	3.5 (2.9–4.1)	2.9 (2.53.4)	
		DE positive	1	4.0 (3.2–5.1)	3.1 (2.9–3.3)	3.1 (2.9–3.3)	
			2	2.1 (1.7–2.7)	1.9 (1.8-2.0)	1.8 (1.7–1.9)	
Males		Any ED	1	10.8 (5.0-23.5)	13.2 (9.1–19.1)	9.1 (6.6–12.4)	
			2	3.3 (1.5–7.3)	4.7 (3.2–6.9)	2.8 (2.0–4.0)	
		Anorexia Nervosa	1	9.0 (2.8–28.7)	14.8 (9.1–24.2)	12.1 (7.9–18.5)	
			2	2.6 (0.8–8.3)	4.9 (2.9–8.1)	3.8 (2.4–6.1)	
		Bulimia Nervosa	1	13.7 (3.3–57.7)	17.9 (8.8–36.3)	8.1 (4.1–15.8)	
			2	4.4 (1.0-18.6)	6.6 (3.1–14.0)	2.8 (1.4–5.7)	
		Binge eating disorder	1	15.2 (5.4–42.5)	11.3 (6.1–20.8)	7.0 (4.1–11.5)	
			2	4.7 (1.7–13.2)	3.9 (2.1–7.4)	2.0 (1.2–3.5)	
		DE positive	1	2.4 (1.6–3.6)	3.5 (3.0-4.1)	3.0 (2.8–3.4)	
			2	1.1 (0.7–1.6)	1.8 (1.5–2.2)	1.6 (1.4–1.8)	
Gender diverse		Any ED	1	3.9 (1.0-15.9)	3.1 (1.6-6.0)	3.1 (1.6–5.8)	
			2	2.7 (0.7–10.8)	2.2 (1.1–4.3)	2.0 (1.0-3.8)	
		Anorexia Nervosa	1	5.8 (1.1–29.6)	3.6 (1.4–9.3)	5.9 (2.2–15.3)	
			2	4.2 (0.8–21.8)	2.8 (1.1–7.4)	3.8 (1.4–10.3)	
		Bulimia Nervosa	1	N/A	6.5 (1.5–28.2)	4.5 (1.0-19.4)	
			2	N/A	4.2 (1.0-18.2)	2.9 (0.7–12.4)	
		Binge eating disorder	1	N/A	2.8 (1.0-7.7)	2.4 (0.9–6.3)	
			2	N/A	2.0 (0.7–5.5)	1.7 (0.6–4.5)	
		DE positive	1	1.4 (0.4-5.0)	3.3 (2.0-5.3)	2.6 (1.7–4.1)	
			2	0.9 (0.3–3.4)	2.4 (1.5-4.0)	1.9 (1.2-3.0)	

**Model** 1: Age adjusted, **model** 2: Adjusted for age and HSCL25 > 2 cut-off

**Reference groups**: DE/ED negative students, respectively

Further, when investigating the association between each item of the EDS-5 scale and suicide attempts, self-harm or suicidal ideation last 12-months, we consistently found the strongest associations, with age- and gender-adjusted ORs 2-4-fold increased, for comfort eating (item 2) as well as feeling guilty about eating (item 3), see Table 4. In addition, students that followed a strict diet (item 4) had a 4-fold increased risk for suicide attempt. Those not satisfied with eating habits had the lowest ORs (ranging from 1.9 to 2.6). Again, adjustment for HSCL-25 (model 2) attenuated the ORs considerably yet remained about 2-fold increased.

**Table 4:** ORs for outcomes (95% CI) and each item of the EDS-5 in 2018 and 2022 (pooled data)

Exposure variables	Adjustment model	Suicide attempts last year	Self-harm last year	Suicidal ideation last year
EDS1*“are you satisfied with your eating habits?”*	1	2.6 (2.2–3.2)	2.2 (2.0-2.4)	2.0 (1.9–2.2)
	2	1.9 (1.5–2.3)	1.6 (1.5–1.8)	
EDS2 “*have you eaten to comfort yourself because you were unhappy?”*	1	3.5 (2.7–4.5)	2.6 (2.4–2.9)	3.2 (3.0-3.4)
	2	1.7 (1.3–2.2)	1.4 (1.3–1.6)	1.6 (1.5–1.8)
EDS3 *“have you felt guilty about eating?”*	1	4.0 (3.3–4.8)	3.3 (3.1–3.5)	3.3 (3.1–3.5)
	2	2.0 (1.7–2.5)	1.9 (1.8-2.0)	1.8 (1.7–1.9)
EDS4 “*have you felt that it was necessary for you to use a strict diet or other eating rituals to control your eating?”*	1	4.3 (3.6–5.3)	2.8 (2.6-3.0)	2.8 (2.6-3.0)
	2	2.5 (2.0-3.1)	1.8 (1.7–1.9)	1.7 (1.5–1.8)
EDS5: “*have you felt you are too fat?”*	1	3.2 (2.7–3.9)	2.8 (2.6–2.9)	2.7 (2.6–2.8)
	2	1.8 (1.5–2.2)	1.7 (1.6–1.9)	1.6 (1.5–1.7)

Model 1: Age and gender adjusted, model 2: Adjusted for age, gender, and HSCL25 > 2 cut-off

**Reference groups**: those responding 1–5 on the EDS

The ROC analysis suggests that, in this sample, the EDS-5 total score showed good discrimination (AUC = 0.84) relative to self-reported past-year ED status; however, this should not be interpreted as a clinical, diagnostic validation. We found consistent results when using the 4.2 cut-off, yielding ORs for suicide attempts: 4.2, self-harm: 3.1 and suicidal ideation: 3.1. This confirmed that a > 4.1 cut-off is very close to the point that maximizes the balance between sensitivity and specificity according to Youden’s index (> 4.2: sensitivity: 80.8%, specificity: 73. 9%).

## Discussion

In a pooled sample of 98 925 Norwegian fulltime students from surveys in 2018 and 2022, we found a consistent pattern of high rates of suicide attempts, self-harm and suicidal ideation last year in female, male and, particularly, gender diverse students with self-reported EDs and DE compared to students without these conditions. Odds ratios (ORs) for the same outcomes in students with self-reported EDs (3- to 13-fold increase) and DE (2- to 4-fold increase) was attenuated, yet remained markedly increased, after adjustment for co-existing anxiety and depression symptoms. ORs were highest among males with self-reported EDs, but precision for males and the gender diverse was limited in several analyses because of small cell counts. Particularly items “comfort eating” and “feeling guilty about eating” on EDS-5 were closely associated with suicide attempts, self-harm and suicidal ideation. Overall, our work is largely in keeping with previous research [16–18, 52, 53], including data from samples of university students [27, 33, 54] and adolescent/emerging adults [34, 55, 56]. Our study broadens existing evidence by widening the perspective regarding exposure (both self-reported diagnostic categories and symptom level) and gender vulnerability (including male and gender-diverse students) in a large national university student sample.

### Suicide attempts, self-harm and suicidal ideation among students with eating disorders and disordered eating

In keeping with our findings, systematic reviews and meta-analyses of individuals with diagnosed and self-reported EDs consistently report high prevalence rates of suicidal behaviours [16–18, 52]. But estimates vary, and direct comparison across these studies is challenging because design, assessment methods and timeframe vary greatly.

The co-occurrence of DE and self-harm was higher in our study than among UK students (4.5–4.9%), also using self-reported ED and DE the previous six months as reference period [27]. A Swedish study reported a 50% lifetime prevalence of self-reported self-harm in students with EDs, and 70% suicide ideation [33], yet lack of gender specific estimates limits direct comparability with our findings. We found slightly higher suicidal ideation rates among female students with EDs compared to a U.S. study total of 690 female college students, where one in four reported suicidal ideation (last two weeks) [57]. Differences in estimates may be caused by different time measures and definitions of self-harm, potential differences in help-seeking behavior or mental health service access, variation between sample and healthcare contexts between countries.

We observed higher frequencies of suicide attempts, self-harm and suicidal ideation among gender diverse students with self-reported ED and DE, compared to their cisgender peers. This in line with a U.S. study of almost 700 gender diverse college students, finding elevated 12-months suicide attempts, self-harm and suicidal ideation in the gender diverse with EDs compared to cisgender with EDs [58], mirroring the high frequencies in gender diverse students in our study, and several other studies of gender diverse students and adolescents [36–38]. A study on over 400 000 college students found that transgender men were significantly more likely to self-report probable EDs than cisgender male peers [35]. Another American study on 5 000 college students found elevated risk for EDs among genderqueer/non-conforming students compared to gender-expansive students. Further, heterosexual or straight trans men had lower odds of ED risk than trans men with minoritized sexual orientations [59]. This demonstrates great variety within gender diverse populations, supporting a more nuanced interpretation of gender diverse results. The high rates among gender diverse might be explained by stigma, bullying, and discrimination, potentially increasing psychological distress and isolation. These stressors are strongly linked to both EDs and suicidality [36–38], placing gender diverse students at particularly high risk. Nevertheless, an American study (*N* = 8 531) found that gender diverse students meeting criteria for EDs were more likely to report chronic ED symptoms and comorbid psychiatric diagnoses compared to heterosexual students and cisgender males, respectively [60], emphasizing the importance of psychiatric comorbidity in sexual- and gender diverse college populations.

In terms of access to care, in the Norwegian context only the largest universities provide extensive student health care services. In turn, this might influence both prevalence and patterns of comorbidity across educational institution in Norway. Previous research using SHoT-data found that services offered by the student welfare organizations seem to play a particularly important role for non-local students and students reporting symptoms of severe mental distress [61], emphasizing the importance of existent student health care services on campus.

### Gender specific associations of eating disorders and disordered eating with suicide attempts, self-harm and suicidal ideation

Our main findings are consistent with previous research pointing to the well-documented association between self-reported EDs and DE, and suicide attempts, self-harm and suicidal ideation among individuals with these conditions [16–18, 52]. Adjustment for anxiety and depression clearly attenuated the ORs, which underscores the importance of considering co-occurring mental health problems when interpreting the results. In student populations, subthreshold ED symptoms aren’t harmless: our findings confirm a strong association to suicidality [37]. Because these eating behaviors are common, and often overlooked, they represent relevant markers of suicidal behaviours on university campuses.

We found the strongest associations of self-reported EDs and DE with suicide attempts, self-harm and suicidal ideation among male students, in line with another Norwegian study on adolescent finding the strongest association between DE and suicidal ideation among males [26]. However, the subgroup analyses should be interpreted with caution. Estimates for male and gender diverse students were derived from relatively small samples and wide CIs, limiting the precision and robustness of the findings. Also, as some of the outcomes are not rare, the ORs should not be interpreted as ratios of proportions or as relative risks. Gender interaction tests provided statistical evidence for higher rates of self-harm and suicidal ideation in males with self-reported EDs compared to females, in contrast with findings from the general population where females usually report higher rates of mental disorders, suicide attempts and self-harm [43, 62]. It is widely accepted that EDs, DE [3], anxiety [63] and depression are all consistently more prevalent among females than males [64–66]. But, while anxiety and depression symptoms in males may have grown increasingly acknowledged, followed by rising prevalence rates of common mental disorders [29], EDs in males have largely gone unnoticed and still represent a “rarity”. It could be assumed that coping with EDs is a lonely process and does not align well with the “male role model”. Consequently, most studies on EDs, suicide attempts, self-harm and suicidal ideation mainly include females [52], with a few exceptions. A study of nearly 15 000 male US college students found that those who reported self-harm or suicidal ideation last 12 months had a 65% increased risk of having comorbid EDs [67]. Further, self-harm in male ED patients was linked to more severe ED symptoms and more affective, interpersonal and impulse-control problems than ED patients without self-harm [68]. Males with EDs experience more stigma, including in the healthcare services, at work, and in their personal lives [69]. Consequently, this affects male help-seeking behaviours, time of diagnosis, interactions with healthcare providers, treatment engagement, and recovery outcomes [69–71]. Therefore, we argue that males with EDs are an especially vulnerable group.

Duration of untreated EDs may be a modifiable factor influencing outcomes [72], as EDs often go undetected [73]. Transdiagnostic approaches may improve scalability, particularly for youth with fluctuating symptoms, within complex family and education systems, by targeting shared mechanisms across comorbidities [74]. A Swedish study indicates that a symptom-focused approach is an effective strategy for identifying subgroups at heightened risk for multiple problem behaviors [75]. With that in mind, our findings highlight a need for targeted interventions.

### Association between each item in the EDS-5 scale and suicide attempts, self-harm and suicidal ideation

The EDS-5 items “comfort eating” and “feeling guilty about eating” were strongly associated with suicide attempts, self-harm and suicidal ideation in our study. We previously found that the same two items also predicted comorbid mental disorders one year after SHoT 2022 more strongly than the three other EDS-5 items (in review). The association between feeling guilty about eating and self-harm and suicidal behaviors could be seen in the context where these feelings might also provoke other unhealthy behaviors. Comfort eating is often related to emotional dysregulation and a need to escape negative affect and rumination [76].

Moreover, maintenance and development of EDs might be related to feelings such as guilt, shame, and disgust [77–79], and potentially contribute to a vicious cycle of EDs through the interplay of negative emotions, dysfunctional thought patterns, and pathological behaviors [80]. Co-occuring EDs and self-harm may share underlying mechanisms and risk factors [81]. Self-harm and EDs could serve as means of affect regulation, punishment, reward, expressing and coping with sexuality, and belonging [82]. Individuals with EDs may use restrictive eating, binge eating, or vomiting as a form of indirect self-harm [83]. In AN, avoidance of social interactions and eating together is common, thereby increasing interpersonal distrust and isolation correlates with guilt [78], while overeating or diverging from strict diets, may lead to compensatory behaviors [84]. Self-criticism may cause self-harm in individuals with DE [85]. Recognizing such patterns is crucial, as they may represent a cycle were comfort eating temporarily alleviates negative emotions, and guilt exacerbates shame and self-criticism, potentially increasing self-harm risk.

### Strengths and limitations

The pooling of data from 2018 to 2022 provided us with a large sample size including 98 925students, which is an important strength of this study. Further, using validated measurements for DE (EDS-5) supports the validity of the study. The results from the ROC-analysis support the robustness of the selected cut-off and indicate that the EDS-5 has acceptable ability to distinguish between students with and without self-reported ED. Our study adds important value by examining both self-reported EDs and DE symptom levels. Lastly, including males and gender diverse students also makes this a novel approach in the field.

Moreover, some limitations should also be considered. First, our sample consisted of more females than males, and the SHoT survey generally has a low response rate, between 31 and 35%, which may affect representativeness and generalizability and limits precision in smaller subgroups. Second, the EDS-5 scale addresses DE in the last 30 days, while the different self-reported ED diagnoses were based on the last 12 months: this should be kept in mind in interpretation of results. Also, self-reported ED diagnosis cannot be compared to studies using clinical diagnostic interviews. From 2018 to 2022, prevalence of DE remained largely the same, while the prevalence of any ED [32] and suicide attempts, self-harm and suicidal ideation increased slightly [30]. The definition of EDs (self-report versus diagnostic diagnosis) and DE differ between studies and should be kept in mind when interpreting results. The results after adjustment of HSCL-25 may represent conservative estimates of the total clinical burden associated with EDs because of an often high diagnostic and symptomatic overlap in psychiatric conditions. Anxiety and depressive symptoms may act as confounders, mediators, or sources of overadjustment, as they may partly lie on the pathway between ED/DE and suicide attempts, self-harm, and suicidal ideation. The possibility that some participants participated in both waves was considered as low, because over a 4-year period, a substantial proportion of the original cohort has completed their degree. However, due to the recruitment procedure in SHoT it was not possible to identify the exact number of participants that participated in both studies. The pooled estimates are average associations across two surveys, but no statistical differences were observed between measures of association in the two surveys separately (appendix A1). Finally, this study’s cross-sectional design rules out the ability to determine temporal and causal relationships.

## Conclusion

Evidence from this study highlights high frequencies of suicide attempts, self-harm and suicidal ideation among students with self-reported EDs and DE, in females, and indicates an association of EDs and DE with suicide attempts, self-harm and suicidal ideation. We found even higher ORs among males and gender diverse students. Adjusting for HSCL-25 still showed ORs between 2 and 4, yet low cell counts in these subgroups limits the robustness of these results. Furthermore, the identification of two specific items on the EDS-5, “Comfort eating” and “feeling guilty about eating”, emerged as potential markers of elevated suicide risk, showing strong associations with suicide attempts, self-harm, and suicidal ideation. These findings offer practical value for clinicians and underscore the importance of considering gender-specific patterns and targeted items to enhance assessment practices.

Our results indicate a high need for clinical services in the student population. Policy measures should guide the administration of university health services, emphasizing the need for structured approaches to early detection and support. There is a need to understand suicidal behaviours in students with EDs and DE, especially male and gender diverse students, as those groups remains understudied and may present with unique risk factors, in addition to excess stigma and help-seeking barriers.

## Supplementary Information

Below is the link to the electronic supplementary material.

Supplementary Material 1

## Data Availability

The data are not available due to ethical considerations observed to maintain the participants’ anonymity in the SHoT survey.

## Abbreviations

AN: Anorexia nervosa
BN: Bulimia nervosa
BED: Binge eating disorder
CIs: Confidence intervals
DE: Disordered eating
EDs: Eating disorders
OR: Odds Ratio
SHoT: The Students’ Health and Wellbeing Study
