# Supplementary material for: Suicide attempts, self-harm and suicidal ideation among Norwegian university students with eating disorders and disordered eating: the SHoT study 2018 and 2022
Source: BMC Psychiatry. 2026 May 30;26:604. doi: 10.1186/s12888-026-08229-0 (PMC13445793; doi:10.1186/s12888-026-08229-0)
Supplement: Supplementary file 1 [file 12888_2026_8229_MOESM1_ESM.docx]

**Table A1:** Students characteristics from SHoT 2018 and 2022, by ED status.

| **Outcome variables** | **Sample by ED status** | |
| --- | --- | --- |
|  | **No ED**  **97.4% (47 469)** | **Any ED**  **2.6% (1 258)** |
| **2018 (N: 48 727)** |  |  |
| Suicide attempts last year | 0.4% (172) | 3.7% (46) |
| Self-harm last year | 3.5% (1 685) | 25.2% (317) |
| Suicidal ideation last year | 6.5% (3 101) | 31.9% (401) |
| **2022 (N: 50 198)** | **No ED**  **96.7% (48 579)** | **Any ED**  **3.3% (1 619)** |
| Suicide attempts last year | 0.4% (197) | 3.6% (58) |
| Self-harm last year | 3.8% (1 868) | 22.6% (366) |
| Suicidal ideation last year | 7.7% (3 764) | 33.0% (535) |

*P-value <.05 for difference between any ED in 2018 and 2022.

**Table A2:** Cross table of students self-reported EDs/DE and outcomes in 2018 and 2022 (pooled).

| **Sex** | **Exposure variables (N)** | **Suicide attempts last year % (N) CI** | **Self-harm last year % (N) CI** | **Suicidal ideation last year % (N) CI** |
| --- | --- | --- | --- | --- |
| **Females** | DE pos (23226) | 1.0% (222) 0.8-1.1 | 8.9% (2062) 8.5-9.3 | 14.0% (3259) 13.6-14.5 |
|  | DE neg (43 157) | 0.24% (104) 0.2-0.3 | 3.1% (1 329) 2.9-3.2 | 5.0% (2 142) 4.7-5.1 |
|  | Any ED (2660) | 3.5% (94) 2.9-4.3 | 23.5% (626) 22.0-25.2 | 31.8% (846) 30.1-33.6 |
|  | No ED (64 008) | 0.4% (234) 0.3-0.4 | 4.3% (2 773) 4.2-4.5 | 7.1% (4 571) 6.9-7.3 |
|  | AN (N: 1519) | 3.9% (59) 3.0-5.0 | 24.9% (378) 22.8-27.1 | 31.3% (476) 29.1-33.7 |
|  | BN (N:1032) | 4.7% (48) 3.8-6.1 | 25.9% (267) 23.3-28.6 | 34.9% (360) 32.0-37.8 |
|  | BED (N:808) | 3.1% (25) 2.1-4.5 | 24.5% (198) 21.7-27.6 | 34.2% (276) 31.0-37.5 |
| **Males** | DE pos (3865) | 0.9% (34) 0.6-1.2 | 5.9% (229) 5.2-6.7 | 16.2% (626) 15.1-17.4 |
|  | DE neg (27 529) | 0.4% (99) 0.3-0.4 | 1.8% (507) 1.7-2.0 | 5.9% (1 613) 5.6-6.1 |
|  | Any ED (166) | 4.2% (7) 2.0-8.6 | 22.9% (38) 17.2-29.9 | 40.4% (67) 33.2-48.0 |
|  | No ED (31 515) | 0.4% (127) 0.3-0.5 | 2.2% (702) 2.1-2.4 | 6.9% (2 187) 6.7-7.2 |
|  | AN (85) | 3.5% (3) 1.1-10.4 | 25.9% (22) 17.7-36.2 | 47.1% (40) 36.8-57.6 |
|  | BN (37) | 5.4% (2) 1.5-17.8 | 29.7% (11) 17.5-45.8 | 37.8% (14) 24.4-53.9 |
|  | BED (65) | 6.2% (4) 2.4-14.8 | 20.0% (13) 12.1-31.3 | 35.4% (23) 24.9-47.5 |
| **Gender diverse** | DE pos (145) | 2.8% (4) 1.0-6.9 | 33.8% (49) 26.6-41.8 | 40.0% (58) 32.4-48.1 |
|  | DE neg (298) | 2.0% (6) 0.9-4.3 | 13.8% (41) 10.3-18.1 | 20.5% (61) 16.3-25.4 |
|  | Any ED (44) | 6.8% (3) 2.3-18.2 | 40.9% (18) 27.7-55.6 | 50.0% (22) 35.8-64.2 |
|  | No ED (399) | 1.8% (7) 0.9-3.6 | 18.0% (72) 14.6-22.1 | 24.3% (97) 20.4-28.8 |
|  | AN (20) | 10.0% (2) 2.8-30.1 | 45.0% (9) 25.8-65.8 | 65.0% (13) 43.3-81.9 |
|  | BN (8) | N/A | 62.5% (5) 30.6-86.3 | 62.5% (5) 30.6-86.3 |
|  | BED (18) | N/A | 38.9% (7) 20.3-61.4 | 44.4% (8) 24.6-66.3 |
